# Supplementary material for: The usage of a modified straight-leg raise neurodynamic test and hamstring flexibility for diagnosis of non-specific low back pain: A cross-sectional study
Source: PLoS One. 2024 May 21;19(5):e0298257. doi: 10.1371/journal.pone.0298257 (PMC11108176; doi:10.1371/journal.pone.0298257)
Supplement: Attachment — Submitted filename: COMMENTS TO THE AUTHORS- PLOS ONE.docx [file pone.0298257.s001.docx]

**COMMENTS TO THE AUTHORS**

The authors conducted significant study to investigate usage of a modified straight-leg raise neurodynamic test and hamstring flexibility to aid in the diagnosis of non-specific low back pain. The manuscript is well-written, and the methodology seems good. However, some comments should be answered before considering this manuscript for publication in PLOS ONE.

1. “Furthermore, hamstring flexibility was also associated with lumbar nerve root compression” ***line 396-397***. This statement contradicts the finding in the result section (Table 2) that showed no significant differences by LBP or impingement. Please clarify.
2. “In this study the majority (80%) of subjects reported having non-specific LBP only,

meaning they did not experience any radiating pain in their legs. Three subjects, however, did report having LBP with radiating pain, which suggests that they may be experiencing some form of nerve compression or irritation that is causing the pain to radiate down their legs” ***line 406- 409***. In our study only 20% (3/15) of our LBP subjects had leg pain,” ***line 453-454***. These details are not reported in the results section, particularly under the characteristics of the subjects. Suggestion that this information be included in the results section.

3. Justify all paragraphs in this manuscript to ensure that information is well organized.
