# Supplementary material for: The usage of a modified straight-leg raise neurodynamic test and hamstring flexibility for diagnosis of non-specific low back pain: A cross-sectional study
Source: PLoS One. 2024 May 21;19(5):e0298257. doi: 10.1371/journal.pone.0298257 (PMC11108176; doi:10.1371/journal.pone.0298257)
Supplement: Attachment — Submitted filename: Review comments on Manuscript Number.doc [file pone.0298257.s002.doc]

**Review comments on Manuscript Number: PONE-D-23-29503. “The usage of a modified straight-leg raise neurodynamic test and hamstring flexibility to aid in the diagnosis of non-specific low back pain"**

**Overall, the idea of research is very interesting to be studied nowadays** and paper is coherently developed. However, there are some comments and suggestions.

General comments the authors need to review the manuscript grammar and editing.

**Title**

- You may consider change the title to [The usage of a modified straight-leg raise neurodynamic test and hamstring flexibility for diagnosis of non-specific low back pain]

**Abstract**

- Don’t use abbreviations in abstract unless you first write the full terms. Correct [mSLR, LBP, HL]
- The methods section should include information about the study sample [number of participants and age]
- It is recommended to write 5- 7 keywords in alphabetical order.

**Introduction**

- Well-structured but needs to be more concise.

**Subjects and methods**

- The age range is relatively wide from 18 to 50. I am afraid that many factors may influence your results

**Statistical analysis**

- Well structured

**Discussion**

- Well structured
