# Supplementary material for: The usage of a modified straight-leg raise neurodynamic test and hamstring flexibility for diagnosis of non-specific low back pain: A cross-sectional study
Source: PLoS One. 2024 May 21;19(5):e0298257. doi: 10.1371/journal.pone.0298257 (PMC11108176; doi:10.1371/journal.pone.0298257)
Supplement: Attachment — Submitted filename: Review comments on Manuscript Number.doc [file pone.0298257.s004.doc]

**Review comments on Manuscript Number: (PONE-D-23-29503R1) entitled’The usage of a modified straight-leg raise neurodynamic test and hamstring flexibility for diagnosis of non-specific low back pain: A cross-sectional study’.**

Overall, this study provides a novel approach.  The idea of research is very interesting, well written and reasonable. I have no comments regarding the manuscript and recommend accepting it.
